# On the Inverse Problem of Binocular 3D Motion Perception

**DOI:** 10.1371/journal.pcbi.1000999

**Published:** 2010-11-18

**Authors:** Martin Lages, Suzanne Heron

**Affiliations:** School of Psychology, University of Glasgow, Glasgow, Scotland; New York University, United States of America

## Abstract

It is shown that existing processing schemes of 3D motion perception such as interocular velocity difference, changing disparity over time, as well as joint encoding of motion and disparity, do not offer a general solution to the inverse optics problem of local binocular 3D motion. Instead we suggest that local velocity constraints in combination with binocular disparity and other depth cues provide a more flexible framework for the solution of the inverse problem. In the context of the aperture problem we derive predictions from two plausible default strategies: (1) the vector normal prefers slow motion in 3D whereas (2) the cyclopean average is based on slow motion in 2D. Predicting perceived motion directions for ambiguous line motion provides an opportunity to distinguish between these strategies of 3D motion processing. Our theoretical results suggest that velocity constraints and disparity from feature tracking are needed to solve the inverse problem of 3D motion perception. It seems plausible that motion and disparity input is processed in parallel and integrated late in the visual processing hierarchy.

## Author Summary

Humans and many other predators have two eyes that are set a short distance apart so that an extensive region of the world is seen simultaneously by both eyes from slightly different points of view. Although the images of the world are essentially two-dimensional, we vividly see the world as three-dimensional. This is true for static as well as dynamic images. Here we elaborate on how the visual system may establish 3D motion perception from local input in the left and right eye. Using tools from analytic geometry we show that existing 3D motion models offer no general solution to the inverse optics problem of 3D motion perception. We suggest a flexible framework of motion and depth processing and suggest default strategies for local 3D motion estimation. Our results on the aperture and inverse problem of 3D motion are likely to stimulate computational, behavioral, and neuroscientific studies because they address the fundamental issue of how 3D motion is represented in the visual system.

## Introduction

The representation of the three-dimensional (3D) external world from two-dimensional (2D) retinal input is a fundamental problem that the visual system has to solve [1]–[4]. This is true for static scenes in 3D as well as for dynamic events in 3D space. For the latter the inverse problem extends to the inference of dynamic events in a 3D world from 2D motion signals projected into the left and right eye. In the following we exclude observer movements and only consider passively observed motion.

Velocity in 3D space is described by motion direction and speed. Motion direction can be measured in terms of azimuth and elevation angle, and motion direction together with speed is conveniently expressed as a 3D motion vector in a cartesian coordinate system. Estimating such a vector locally is highly desirable for a visual system because the representation of local estimates in a dense vector field provides the basis for the perception of 3D object motion, that is direction and speed of moving objects. This information is essential for interpreting events as well as planning and executing actions in a dynamic environment.

**Figure 1:**
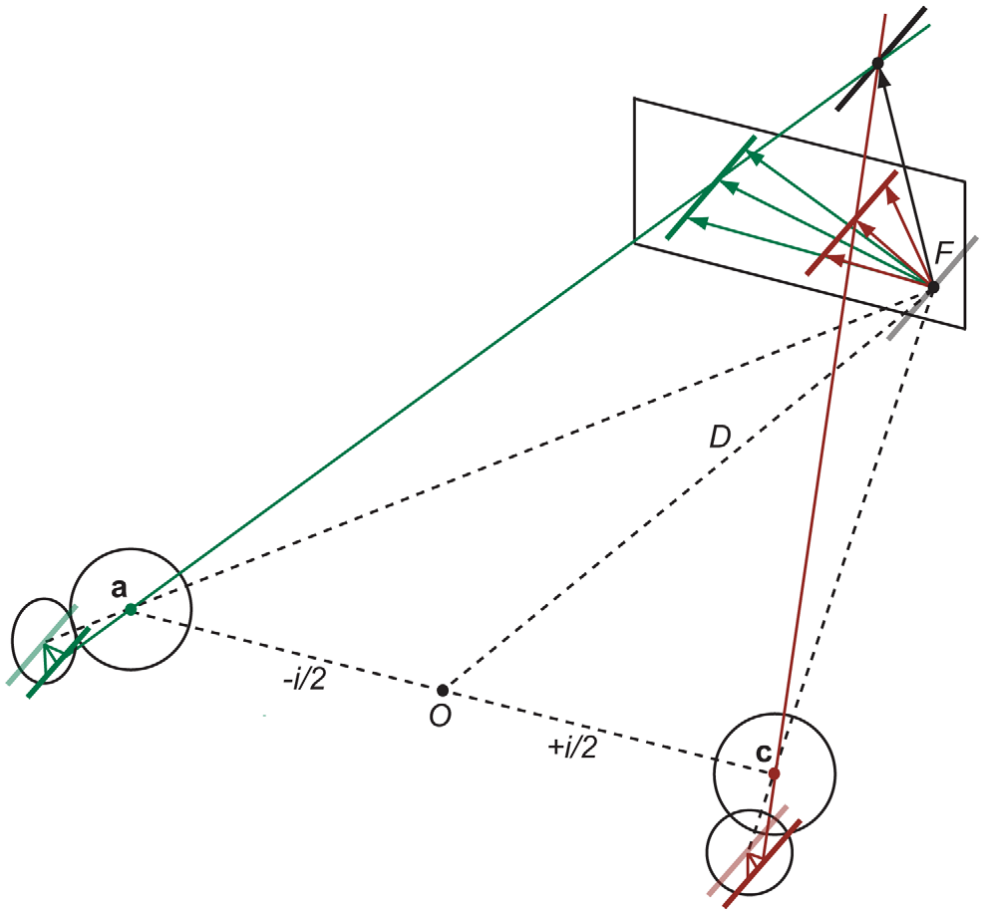
Illustration of the aperture problem of 3D motion with projections of an oriented line or contour moving in depth. The left and right eye with nodal points **a** and **c**, separated by interocular distance *i*, are verged on a fixation point *F* at viewing distance *D*. If an oriented stimulus (diagonal line) moves from the fixation point to a new position in depth along a known trajectory (black arrow) then perspective projection of the line stimulus onto local areas on the retinae or a fronto-parallel screen creates 2D aperture problems for the left and right eye (green and brown arrows).

If a single moving point, corner or other unique feature serves as binocular input then intersection of constraint lines or triangulation together with a starting point provides a straightforward and unique geometrical solution to the inverse problem in a binocular viewing geometry (see Methods and Fig. 1 for an illustration). If, however, the moving stimulus has spatial extent, such as an edge, contour, or line inside a circular aperture [5] then local motion direction in corresponding receptive fields of the left and right eye remains ambiguous and additional constraints are needed to solve the aperture and inverse problem in 3D.

The inverse optics and the aperture problem are well-known problems in computational vision, especially in the context of stereo [3], [6], structure from motion [7], and optic flow [8]. Gradient constraint methods belong to the most widely used techniques of optic-flow computation from image sequences. They can be divided into local area-based [9] and into more global optic flow methods [10]. Both techniques employ brightness constancy and smoothness constraints in the image to estimate velocity in an over-determined equation system. It is important to note that optical flow only provides a constraint in the direction of the image gradient, the normal component of the optical flow. As a consequence some form of regularization or smoothing is needed.

Similar techniques in terms of error minimization and regularization have been offered for 3D stereo-motion detection [11]–[13]. Essentially these algorithms extend processing principles of 2D optic flow to 3D scene flow.

Computational studies on 3D motion algorithms are usually concerned with fast and efficient encoding when tested against ground truth. Here we are less concerned with the efficiency or robustness of a particular implementation. Instead we want to understand and predict behavioral characteristics of human 3D motion perception. 2D motion perception has been extensively researched in the context of the 2D aperture problem [14]–[16] but there is a surprising lack of studies on the aperture problem and 3D motion perception.

Any physiologically plausible solution to the inverse 3D motion problem has to rely on binocular sampling of local spatio-temporal information. There are at least three known cell types in early visual cortex that may be involved in local encoding of 3D motion: simple and complex motion detecting cells [17]–[20], binocular disparity detecting cells [21] sampled over time, and joint motion and disparity detecting cells [22]–[24].

It is therefore not surprising that three approaches to binocular 3D motion perception have emerged in the literature: Interocular velocity difference (IOVD), changing disparity over time (CDOT), and joint encoding of motion and disparity (JEMD).

These three approaches have generated an extensive body of research but psychophysical results have been inconclusive and the nature of 3D motion processing remains an unresolved issue [25], [26]. Despite the wealth of empirical studies on motion in depth there is a lack of studies on true 3D motion stimuli. Previous psychophysical and neurophysiological studies typically employ stimulus dots with unambiguous motion direction or fronto-parallel random-dot surfaces moving in depth. The aperture problem and local motion encoding however, which features so prominently in 2D motion perception [14]–[16] has been neglected in the study of 3D motion perception.

Large and persistent perceptual bias has been found for dot stimuli with unambiguous motion direction [27]–[29] suggesting processing strategies that are different from the three main processing models [28]–[30]. It seems promising to investigate local motion stimuli with ambiguous motion direction such as a line or contour moving inside a circular aperture [31] because they relate to local encoding [17]–[24] and may reveal principles of 3D motion processing [32].

The aim of this paper is to evaluate existing models of 3D motion perception and to gain a better understanding of binocular 3D motion perception. First, we show that existing models of 3D motion perception are insufficient to solve the inverse problem of binocular 3D motion. Second, we establish velocity constraints in a binocular viewing geometry and demonstrate that additional information is necessary to disambiguate local velocity constraints and to derive a velocity estimate. Third, we compare two default strategies of perceived 3D motion when local motion direction is ambiguous. It is shown that critical stimulus conditions exist that can help to determine whether 3D motion perception favors slow 3D motion or averaged cyclopean motion.

## Results

In the following we summarize shortcomings for each of the three main approaches to binocular 3D motion perception in terms of stereo and motion correspondence, 3D motion direction, and speed. We also provide a counterexample to illustrate the limitations of each approach.

### Interocular velocity difference (IOVD)

This influential processing model assumes that monocular spatio-temporal differentiation or motion detection [33] is followed by a difference computation between velocities in the left and right eye [34]–[36]. The difference or ratio between monocular motion vectors in each eye, usually in a viewing geometry where interocular separation *i* and viewing distance *D* is known, provides an estimate of motion direction in terms of azimuth angle only.

We argue that the standard IOVD model [29], [37]–[40] is incomplete and ill-posed if we consider local motion encoding and the aperture problem. In the following the limitations of the IOVD model are illustrated.

#### Stereo correspondence

The first limitation is easily overlooked: IOVD assumes stereo correspondence between motion in the left and right eye when estimating 3D motion trajectory. The model does not specify which motion vector in the left eye should correspond to which motion vector in the right eye before computing a velocity difference. If there is only a single motion vector in the left and right eye then establishing a stereo correspondence appears trivial since there are only two positions in the left and right eye that signal dynamic information. Nevertheless, stereo correspondence is a necessary pre-requisite of IOVD processing which quickly becomes challenging if we consider multiple stimuli that excite not only one but many local motion detectors in the left and right eye. It is concluded that without explicit stereo correspondence between local motion detectors the IOVD model is incomplete.

#### 3D motion direction

**Figure 2:**
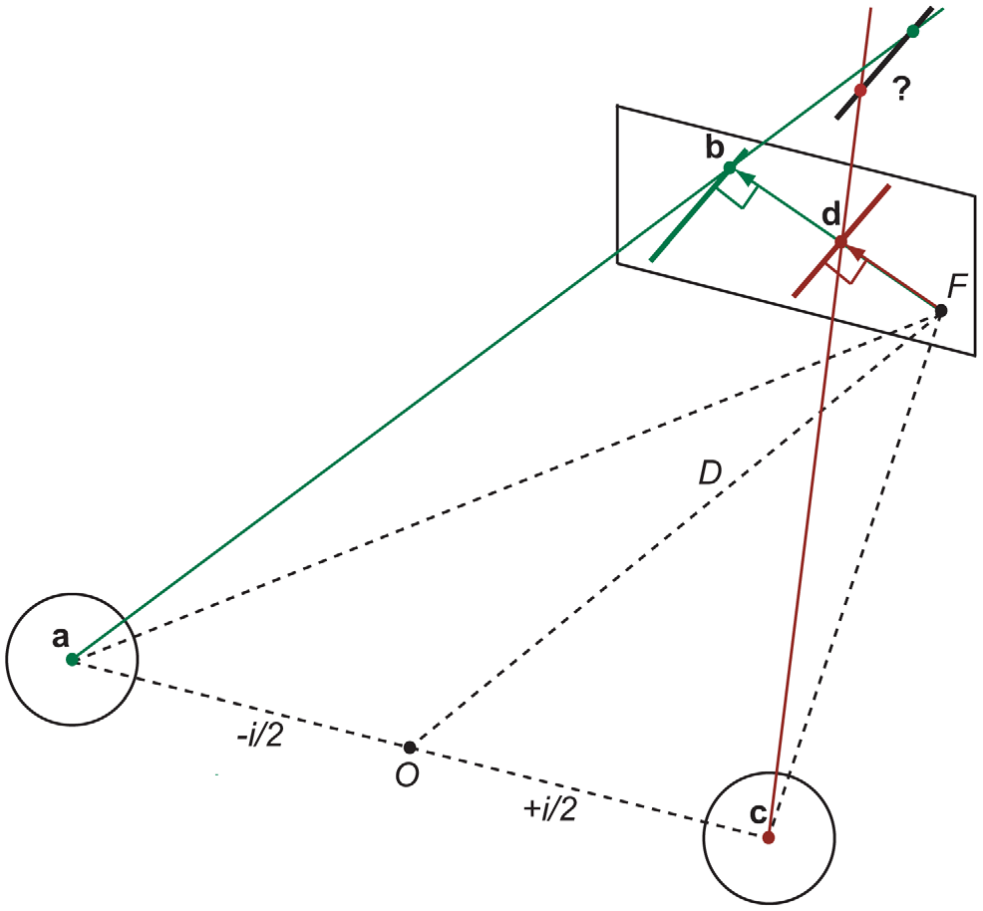
Inverse projection of constraint lines preferring slow 2D motion in the left and right eye. Constraint lines through projection point **b** and **d** do not intersect and 3D motion cannot be determined (see text for details).

The second problem concerns 3D motion trajectories with arbitrary azimuth and elevation angles. Consider a local contour with spatial extent such as an oriented line inside a circular aperture so that the endpoints of the line are occluded. This is known as the aperture problem in stereopsis [5], [41]. If an observer maintains fixation at close or moderate viewing distance then the oriented line stimulus projects differently onto the left and right retina (see Fig. 2 for an illustration with projections onto a single fronto-parallel plane). When the oriented line moves horizontally in depth at a given azimuth angle then local motion detectors tuned to different speeds respond optimally to motion normal (perpendicular) to the orientation of the line. If the normal in the left and right eye serves as a default strategy for the aperture problem in 2D [14], [16] then these vectors may have different lengths (as well as orientations if the line or edge is oriented in depth). Inverse perspective projection of the retinal motion vectors reveals that the velocity constraint lines are skew and an intersection of line constraints (IOC) does not exist. In fact, an intersection only exists if the following constraint for the motion vector in the left and right eye holds (see Methods):
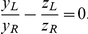
(If the image planes are fronto-parallel so that 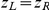 then the condition is simply 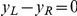). However, this constraint is easily violated as illustrated in Fig. 2 and Counterexample 1 below.

#### Speed

It is worth pointing out that IOVD offers no true estimate of 3D speed. This is surprising because the model is based on spatial-temporal or speed-tuned motion detectors. The problem arises because computing motion trajectory without a constraint in depth does not solve the inverse problem. As a consequence speed is typically approximated by motion in depth along the line of sight [37].

#### Counterexample 1

If an edge or line tilted from horizontal by 0<*θ*<90° moves in depth at a fixed azimuth angle so that horizontal translations of the projected images into the left and right eye are unequal 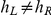, it follows from basic trigonometry that the local motion vectors normal to the oriented line have *y*-co-ordinates 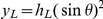 and 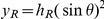, thus 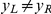 (see Fig. 2 and Methods).

Another violation occurs when the line is slanted in depth and projects with different orientations into the left and right eye. The resulting misalignment on the *y*-axis between motion vectors in the left and right eye is reminiscent of vertical disparity and the induced effect [42], [43] with vertical disparity increasing over time. The stereo system can reconstruct depth from input with orientation disparity and even vertical disparity [44] but it seems unlikely that the binocular motion system can establish similar stereo correspondences.

It is concluded that the IOVD model is incomplete and easily leads to ill-posed inverse problems. These limitations are difficult to resolve within a motion processing system and point to contributions from disparity or depth processing.

### Changing disparity over time (CDOT)

This alternative processing scheme uses disparity input and monitors changing disparity over time (CDOT). Disparity between the left and right image is detected [45] and changes over time give rise to motion-in-depth perception [46]–[49]. We argue that this approach also has limitations when the inverse problem of local 3D motion is considered.

#### Motion correspondence

Assuming CDOT can always establish a suitable stereo correspondence between features including lines [5], [41] then the model still needs to resolve the motion correspondence problem. It needs to integrate disparity not only over time but also over 3D position to establish a 3D motion trajectory. Although this may be possible for a global feature tracking system it is unclear how CDOT arrives at estimates of local 3D motion.

#### 3D motion direction

Detecting local disparity change alone is insufficient to determine an arbitrary 3D trajectory. CDOT has difficulties to recover arbitrary 3D motion direction because only motion-in-depth along the line of sight is well defined. 3D motion direction in terms of arbitrary azimuth and elevation requires a later global mechanism that has to solve the inverse problem by tracking not only disparity over time but also position in 3D space over time.

#### Speed

As a consequence the rate of change of disparity provides a speed estimate for motion-in-depth along the line of sight but not for arbitrary 3D motion trajectories.

#### Counterexample 2

In the context of local surface motion consider a horizontally slanted surface moving to the left or right behind a circular aperture. Without corners or other unique features CDOT can only detect local motion in depth along the line of sight. Similarly in the context of local line motion, the inverse problem remains ill posed for a local edge or line moving on a slanted surface because additional motion constraints are needed to determine a 3D motion direction.

In summary, CDOT does not provide a general solution to the inverse problem of local 3D motion because it lacks information on motion direction. Even though CDOT is capable of extracting stereo correspondences over time, additional motion constraints are needed to represent arbitrary motion trajectories in 3D space.

### Joint encoding of motion and disparity (JEMD)

This approach postulates that early binocular cells are both motion and disparity selective and physiological evidence for the existence of such cells was found in cat striate cortex [22] and monkey V1 [50] (see however [51]). Model cells in this hybrid approach extract motion and disparity energy from local stimulation. A read-out from population activity and population decoding is needed to explain global 3D motion phenomena such as transparent motion and Pulfrich-like effects [52], [53]. Although JEMD is physiologically plausible it shares two problems with IOVD.

#### 3D motion direction

Similar to cells tuned to binocular motion, model cells of JEMD prefer corresponding velocities in the left and right eye. Therefore a binocular model cell can only establish a 2D fronto-parallel velocity constraint at a given depth. Model cell activity remains ambiguous because it can be the result of local disparity or motion input [54]. A later processing stage, possibly at the level of human V5/MT [55] needs to read out population cell activities across positions and depth planes and has to approximate global 3D motion. Similar to CDOT, the model defers the inverse problem to a later global processing stage.

#### Speed

Again, similar to IOVD and CDOT, JEMD provides no local 3D speed estimate. It also has to rely on sampling across depth planes in a population of cells in order to approximate speed.

#### Counterexample 3

Consider local 3D motion with unequal velocities in the left and right eye but the same average velocity, e.g. diagonal trajectories to the front and back through the same point in depth. JEMD has no mechanism to discriminate between these local 3D trajectories when monitoring binocular cell activity across depth planes in a given temporal window.

In the following we introduce general velocity constraints for 3D motion and suggest two default strategies of 3D motion perception that are based on different processing principles (see Methods for details).

### Velocity constraints and two default strategies

Which constraints does the visual system use to solve the inverse as well as aperture problem for local 3D line motion where endpoints are invisible or occluded? This is a critical question because it is linked to local motion encoding and the possible contribution from depth processing.

**Figure 3:**
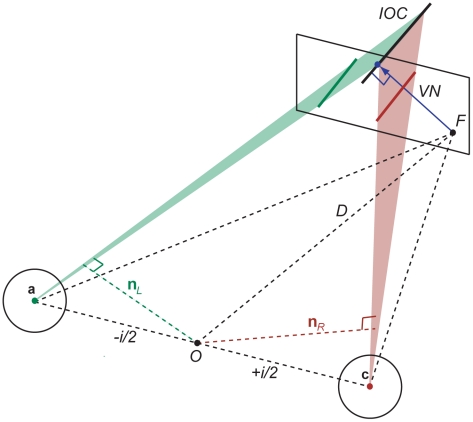
Illustration of vector normal (VN) as a default strategy for local 3D motion perception (see text for details). The intersection of constraint planes (IOC) together with the assumption of slow motion describes the shortest vector in 3D space (blue arrow) that fulfills the velocity constraints.

The 3D motion system may establish constraint planes rather than constraint lines to capture all possible motion directions of a contour or edge, including motion in the direction of the edge's orientation. Geometrically the intersection of two constraint planes in a given binocular viewing geometry defines a constraint line oriented in 3D velocity space (see Fig. 3 and Methods).

We suggest that in analogy to 2D motion perception [15], [56] tracking of features in depth coupled with binocular velocity constraints from motion processing provides a flexible strategy to disambiguate 3D motion direction and to solve the inverse problem of 3D motion perception.

But which principles or constraints are used? Does the binocular motion system prefer slow 3D motion or averaged 2D motion? Does it solve stereo correspondence before establishing binocular velocity constraints or does it average 2D velocity constraints from the left and right eye before it solves stereo correspondence? We derive predictions for two alternative strategies to address these questions.

#### Vector normal (VN)

Velocity constraints in the left and right eye provide velocity constraint planes in 3D velocity space. In Fig. 3 they are illustrated as translucent green and brown triangles in a binocular viewing geometry. The intersection of constraint planes defines a velocity constraint line in 3D that also describes the true end-position of the moving line or contour (black line). The vector or line normal from the oriented constraint line to the starting point gives a default 3D motion estimate (blue arrow). It is the shortest distance in 3D velocity space and denotes the slowest motion vector that fulfills both constraints. Note that this strategy requires that the 3D motion system has established some stereo correspondence so that the intersection of constraints as well as the vector normal can be found in 3D velocity space.

The VN strategy is a generalization of the vector normal and IOC in 2D [15] and it is related to area-based regression and gradient constraint models [9] where the local brightness constancy constraint ensures a default solution that is normal to the orientation of image intensity.

#### Cyclopean average (CA)

**Figure 4:**
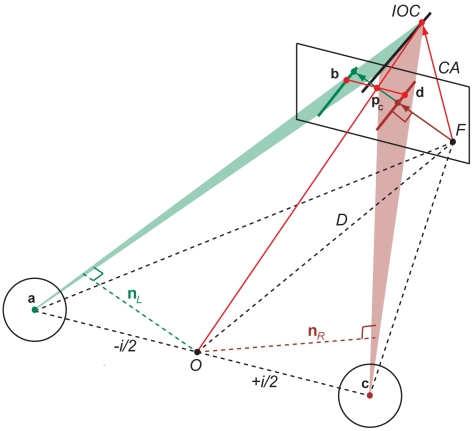
Illustration of cyclopean average (CA) as a default strategy for local 3D motion perception (see text for details). Combining the cyclopean velocity constraint with horizontal disparity determines a vector in 3D space (red arrow) with average monocular velocity.

If the motion system computes slow 2D motion independently in the left and right eye then the cyclopean average provides an alternative velocity constraint [27], [57]. Averaging of monocular constraints increases robustness of the motion signal at the expense of binocular disparity information. Thus, a cyclopean average constrains velocity but gives no default estimate of velocity. However, if we attach (dynamic) disparity to the cyclopean average then the CA provides a default estimate of 3D velocity (see Methods and Fig. 4).

The CA strategy is a generalized version of the vector average strategy for 2D motion [58] and can be linked to computational models of 3D motion that use global gradient and smoothness constraints [10]. These global models amount to computing the average flow vector in the neighborhood of each point and refining the scene flow vector by the residual of the average flow vectors in the neighborhood. Interestingly, tracking the two intersection points or T junctions of a moving line with a circular aperture in the left and right eye and averaging the resulting vectors gives predictions that are equivalent to the CA strategy.

#### Predictions for VN and CA strategy

We use the Vector Normal (VN) and Cyclopean Average (CA) as default strategies to predict 3D velocity of an oriented line or contour moving in depth inside a circular aperture.

**Figure 5:**
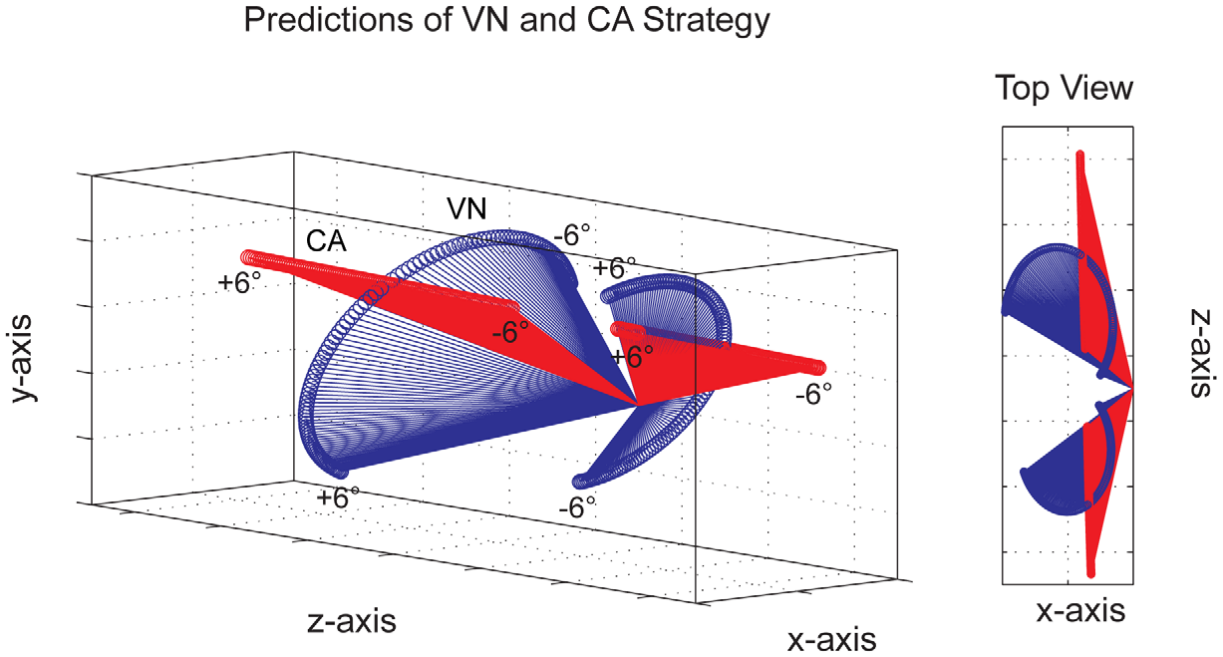
Velocity predictions of vector normal (VN, blue) and cyclopean average (CA, red) as default strategies of perception of local 3D line motion. Predictions for an oriented stimulus line moving on a fixed trajectory to the front left and to the back left are shown. Predicted velocities show characteristic differences when the moving stimulus line or contour is slanted in depth (range of orientation disparities between −6° to +6°).

The 3D plot in Fig. 5 shows predictions of the VN strategy (blue) and the CA strategy (red) for a diagonal line stimulus moving on two trajectories in depth at a viewing distance *D* = 57 cm and interocular distance of *i* = 6.5 cm. The line stimulus has a trajectory to the front and left with azimuth +57.2 deg and elevation 0 deg, and a trajectory to the back and left with azimuth −57.2 deg and elevation 0 deg. Azimuth and elevation of 0 deg denotes a horizontal and fronto-parallel trajectory to the left. The starting point of each trajectory is the origin of the vector fields in the 3D plot. An open circle denotes the endpoint of a predicted motion vector. For each default strategy and stimulus trajectory a field of 120 vectors are shown with orientation disparity of the line stimulus ranging from −6° to +6° in steps of 0.1°. Orientation disparity changes perceived slant of the diagonal line so that at −6° the bottom-half of the line is slanted away from the observer and the top-half is slanted towards the observer.

If the diagonal line is fronto-parallel and has zero orientation disparity both strategies make equivalent predictions (intersection of red and blue vector fields in Fig. 5). If, however, the stimulus line has orientation disparity and is slanted in depth then predictions clearly discriminate between the two strategies. The VN strategy always finds the shortest vector between starting point and moving line so that velocity predictions approximate a semi-circle for changing orientation disparity. Please note that for the VN predictions the sign of orientation disparity reverses for the stimulus trajectory to the front and back. The CA strategy on the other hand computes an average vector and as a consequence the endpoints of the predictions approximate a velocity constraint line through the cyclopean origin.

In a first experiment using a psychophysical matching task we measured the perceived 3D motion direction of an oriented line moving behind a circular aperture. Preliminary results from four observers indicate VN as the default strategy. Perceptual bias from depth processing reduced perceived slant of the stimulus line and this also affected motion direction [30].

## Discussion

IOVD and CDOT are extreme models because they are based on either motion or disparity input. IOVD excludes contributions from binocular disparity processing but requires early stereo correspondence. It does not solve the inverse problem for local 3D line motion because it is confined to 3D motion in the *x*- or *z*-plane.

CDOT on the other hand excludes contributions from motion processing and therefore has problems to establish motion correspondence and direction. Without further assumptions it is confined to motion in depth along the line of sight.

If either motion or disparity input determines 3D motion perception then processing of any additional input needs to be disengaged or silenced. Instead, the visual system may take advantage of motion and disparity input [59], [60] as well as additional cues. Here we favor parallel processing and late integration over early joint encoding because the inverse problem for local 3D motion remains ill posed for JEMD and a population read-out needs to be specified to approximate global 3D motion at a later stage.

Combining global disparity or depth information with local velocity constraints at a later stage solves the inverse problem of local 3D motion and provides a flexible scheme that can exploit intermediate depth processing such as relative and orientation disparity in V2 and V4 [44], [61]. Velocity constraints may be processed in the ventral stream and binocular disparity together with other depth cues in the dorsal stream [62]. It seems anatomically and neurophysiologically plausible that integration of motion and disparity occurs late in subregions of human V5/MT [55], [63]–[65] if not in areas beyond V5/MT [66].

What enables the visual system to instantaneously perceive 3D motion and to infer direction and speed of a moving object? It seems likely that the visual system exploits many cues to make this difficult inference as reliable and veridical as possible and the diverse set of effective local and global cues in psychophysical studies [59], [67] already points at late integration within the visual processing hierarchy [62], [65], [66].

More specifically, we suggest that binocular 3D motion perception may be based on parallel motion and depth processing. Thereby motion processing captures local spatio-temporal constraints in the scene whereas depth processing provides a global and dynamic depth map that helps to disambiguate motion direction and to maintain a detailed spatial representation of the scene. Late integration of motion and disparity constraints in combination with other cues can solve the inverse problem of local 3D motion and allows the visual system to remain flexible when binding and segmenting local inputs from different processing stages into a global 3D motion percept. Parallel processing and late integration may explain why, compared to 2D motion perception, 3D motion perception shows reduced spatio-temporal tuning characteristics [68], [69] and why motion perception can retain relatively fine spatial detail. The combination of local motion constraints with a global dynamic depth map from higher-order features would also explain the perception of different types of non-linear motion, such as non-rigid and 2^nd^ order motion.

The notion of parallel pathways feeding functionally different aspects of motion perception into a later stage is not new and has been advanced in the context of 2D motion direction and speed perception [70], [71], 2D pattern motion [15], [56], [58], eye movements [72], [73], and the processing of higher order motion [74], [75] but was not often addressed in the context of binocular 3D motion perception [75], [76].

Considering the ill-posed inverse problem of existing approaches and the under-determined characteristics of local binocular motion constraints, parallel processing and late integration of motion and disparity as well as other cues appears particularly convincing because solving the inverse problem for local 3D motion adds a functional significant aspect to the notion of parallel streams of dynamic disparity and motion processing. It will require considerable efforts to unravel the entire process but recent developments in the framework of Bayesian inference [28], [29], [56] look promising to extend the geometric considerations given here.

## Methods

In the following we assume a fixed binocular viewing geometry with the cyclopean origin 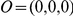 centered ±*i/2* between the nodal points of the left and right eye and the eyes verged on a fixation point straight ahead at viewing distance *D* (see Fig. 1). More complicated geometries arise if we take into account version, cyclovergence, and cyclotorsion of the eyes [77], [78]. For the sake of simplicity we ignore the non-linear aspects of visual space [79] and represent perceived 3D motion as a linear vector in a three-dimensional Euclidean space where the fixation point is also the starting point of the motion stimulus.

Since we are not concerned about particular algorithms and their implementation, results are given in terms of analytic geometry [80], [81].

### Intersection of constraint lines

If the eyes remain verged on a fixation point in a binocular viewing geometry then the constraint line in the left and right eye can be defined by pairs of points 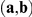 and 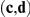, respectively. The nodal point in the left eye 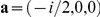 and a projection point 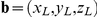 of the motion vector on the left retina define a constraint line for the left eye. Similarly, points 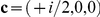 and 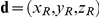 determine a constraint line in the right eye. The corresponding vector directions are given by
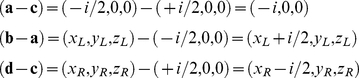
(1)Each constraint line can expressed by a pair of points 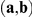 and 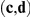 together with scalar *t*:
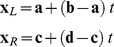
(2)The two lines intersect for
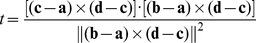
(3)if and only if
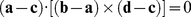
(4)where  is the scalar product also called the dot product, × denotes the cross product, and 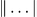 the norm of a vector. Otherwise, the two lines are skew, and the inverse problem is ill posed.

We can exclude the trivial case 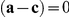 because the two eyes are separated by 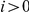. We also exclude the special case where the cross product is zero because the motion vectors in the left and right eye are identical or opposite.

The cross product in (4) can be written as
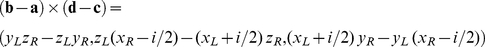
(5)Since 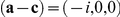 in Eq. (4) we are only concerned with the product 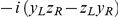 which equals zero if and only if
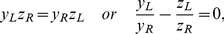
(6)The ratio of *z* co-ordinates on the right-hand side may be different from 1 as a result of eye vergence and the left-hand side reflects the corresponding ratio of vertical displacements.

In the following we consider the simpler case of projections onto a fronto-parallel screen (coplanar retinae) at a fixed viewing distance *D* (see Fig. 2). In this case epipolar lines are horizontal with equivalent co-ordinates 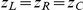 on the *z*-axis.

Again, since 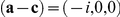 in (4) we only have to evaluate 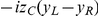 which is zero if and only if:
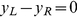
(7)For an intersection to exist the left and right eye motion vector must have equivalent horizontal *y* co-ordinates or zero vertical disparity.

### Intersection of constraint planes

Monocular line motion defines a constraint plane with three points: the nodal point of an eye and two points defining the end position of the projected line (see Fig. 3). In order to find the intersection of the left and right eye constraint plane we use the plane normal in the left and right eye. If the two planes are specified in Hessian normal form
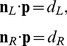
(8)where  is again the dot product, 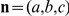 is a vector describing the surface normal to a plane, 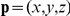 is a vector representing all points on the plane, and *d* is a scalar.

We need to check whether the constraint planes are parallel or coincident, that is if
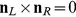
(9)before we can determine their intersection. The equation for the intersection of the two constraint planes is a line here written as
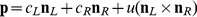
(10)where *u* is a free parameter. Taking the dot product of the above with each plane normal gives two equations with unknown scalars *c_L_* and *c_R_*.
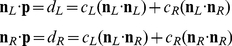
(11)Solving the two equations for *c_L_* and *c_R_* gives
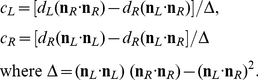
(12)Inserting *c_L_* and *c_R_* in (10) determines the intersection of constraints or constraint line **p**.

In analogy to the 2D aperture problem and the intersection of constraints we can now define two plausible strategies for solving the 3D aperture problem:

### Vector normal (VN)

The shortest distance in 3-D (velocity) space between the starting point 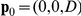 of the stimulus line and the constraint line 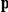 is the line or vector normal through point 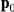. In order to determine the intersection point of the vector normal with the constraint line we pick two arbitrary points 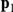 and 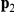 on intersection constraint line 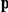 by choosing a scalar *u* (e.g., 0.5).
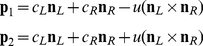
(13)Together with point 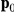 we can compute scalar 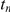 as
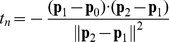
(14)which determines the closest intersection point 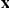 on the constraint line:
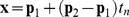
(15)


### Cyclopean average (CA)

We can define a cyclopean constraint line in terms of the cyclopean origin 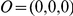 and projection point 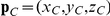 on a fronto-parallel screen where 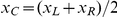 and 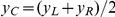 are the averages of the 2D normal co-ordinates for the left and right eye projections.

If we measure disparity 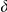 at the same retinal coordinates as the horizontal offset between the left and right eye anchored at position 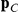 then we can define new points **b** with 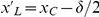 and **d** with 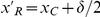. (Alternatively, we may establish an epipolar or more sophisticated disparity constraint.) The resulting two points together with the corresponding nodal points **a** and **c** define two constraint lines as in (2), one for the left and the other for the right eye. By inserting the new co-ordinates from above into (4) it is easy to see that condition (6) holds and the scalar for the intersection of lines can be found as in (3).

### Transformation into spherical co-ordinates

The intersection 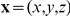 in cartesian co-ordinates can be transformed into spherical co-ordinates 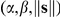 using vectors 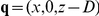 and 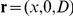 to determine azimuth *α* in the horizontal plane
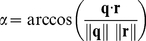
(16)Similarly, for base vectors 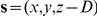 and 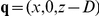 elevation *β* is given by
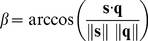
(17)Speed in 3D space is equivalent to the norm of vector **s** written as 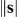.
